# Relationships between dental personnel and non-dental primary health care providers in rural and remote Queensland, Australia: dental perspectives

**DOI:** 10.1186/s12903-017-0389-y

**Published:** 2017-06-19

**Authors:** Jackie Stuart, Ha Hoang, Len Crocombe, Tony Barnett

**Affiliations:** Centre for Research Excellence in Primary Oral Health Care, Centre for Rural Health, School of Health Sciences, University of Tasmania, Locked Bag 1322, Launceston, TAS 7250 Australia

**Keywords:** Collaboration, Dental practitioners, Interprofessional relationships, Primary care providers, Oral health, Rural and remote health

## Abstract

**Background:**

Collaboration between dental practitioners and non-dental primary care providers has the potential to improve oral health care for people in rural and remote communities, where access to oral health services is limited. However, there is limited research on collaboration between these professional disciplines. The purpose of this paper was to explore the relationships between dental practitioners and non-dental primary care providers from rural and remote areas of Queensland and to identify strategies that could improve collaboration between these disciplines from the perspective of dental participants.

**Methods:**

Semi-structured interviews were conducted between 2013 and 2015 with visiting, local and regional dental practitioners (*n* = 12) who had provided dental services to patients from eight rural and remote Queensland communities that did not have a resident dentist. Participants were purposely recruited through a snow ball sampling technique. Interview data were analysed using thematic analysis with the assistance of QSR Nvivo v.10.

**Results:**

Four major themes emerged from the data: (1) Communication between dental practitioners and rural primary care providers; (2) Relationships between dental and primary care providers; (3) Maintenance of professional dualism; (4) Strategies to improve interprofessional relationships (with subthemes: face to face meetings; utilisation of technology; oral health training for primary care providers; and having a community based oral health contact person). Participants observed that there was a lack of communication between the dental providers who saw patients from these rural communities and the primary care providers who worked in each community. This was attributed to poor communication, the high turnover of staff and the siloed behaviours of some practitioners. Visiting dental practitioners were likely to have stronger professional relationships with hospital nursing, administrative and allied health care staff who were often long term residents of the community.

**Conclusions:**

The findings suggest that there was little relationship between the dental personnel and primary care providers. Interprofessional collaboration between dental care providers and non-dental rural primary care providers in the rural and remote communities sampled could be improved by having regular face to face meetings between practitioners from across the health disciplines, providing oral health education to primary care providers, establishing and maintaining effective communication and referral pathways, and exploring a greater role for tele-dentistry.

## Background

Oral health services in Australia are provided by both government and private sectors [1]. Government provides low cost or fully subsidised oral health services for children up to 18 years old and low income adults [1]. Rural and remote Australians have poorer oral health [2] than those living in cities. Rural communities in Australia are often widely scattered and often, do not have a sufficient population to support a full-time dentist. In the lack of a dental practice in a rural community, rural and remote residents may be serviced by public and/or private visiting oral health services [3]. People may also travel to larger population centres to access dental services and incur additional costs associated with the travel, time off work [4, 5], arranging alternative care for dependents, and return visits to the dentist if required for optimal treatment [6]. When there is a lack of oral health services, people with an acute oral health problem may present to medical doctors [7], hospital emergency departments [7–9], pharmacies [10] or to an Aboriginal Health Centre [11, 12]. Non-dental practitioners are usually able to provide only temporary relief of symptoms and referral rather than definitive treatment [7, 10, 13] due to lack of oral health training [13], safety and scope of practice considerations. Consequently, both dental and rural primary care providers may be involved in providing oral health care advice and treatment to rural and remote communities.

Interprofessional collaboration is the process in which different disciplines work together to improve the quality of patient centred care [14] . Collaboration between dental care providers and rural primary care providers has the potential to improve oral health care for rural people who often have limited access to oral health services and may present to primary care providers with oral health problems for advice and treatment. Furthermore, medical conditions including diabetes, cardiovascular disease, stroke and adverse pregnancy outcomes have been associated with oral diseases such as gingivitis and periodontitis [15–20]. Many patients with co-existing dental and medical issues would benefit from a health care plan developed through co-operation by medical and dental health care professionals [21–24]. Acknowledging this interrelationship, the World Health Assembly in 2007 proposed that oral health care and chronic disease prevention programs should be better integrated [25]. It is important to investigate how these two disciplines work together to provide oral health care to rural people. However, there is limited research on such collaboration.

The purpose of this paper was to explore the relationships between dental practitioners and non-dental primary care providers in the provision of oral health services to rural and remote Queensland communities and strategies that could improve collaboration between these disciplines from the perspective of the dental personnel.

## Methods

This study was part of a larger project, which investigated the relationship between dental practitioners and primary care networks in rural and remote areas of Queensland (QLD), South Australia and Tasmania, Australia. The state’s chief dental officer was invited to identify rural and remote communities in which oral health care was a significant problem, and where there was at least one general medical practice, a health care facility and a pharmacy, but no resident dentist. Eight rural and remote communities in QLD were identified for inclusion in this study (Table 1). Each of these communities experienced varying and often sporadic levels of oral health service provision. These services included: three monthly government dental practitioner visits to some communities, occasional fly in/fly out private dental practitioner services, once yearly mobile dental services for Indigenous patients, sporadic school dental therapist visits, and, more recently, a free mobile dental service associated with the Royal Flying Doctor Service and mining company the QCoal Group. Patients from all communities were encouraged to travel to larger regional centres for oral health care in the absence of a resident dentist. Patients with a health care card were eligible for treatment from government dental clinics but were often required to find their own transport. The local government in some outreach communities allocated funding for bus fares, to transport these patients to the government dental clinic. Vouchers were also provided to patients who could not receive timely treatment from the government dental practitioner, enabling them to attend regional private dental practitioners.

**Table 1:** Characteristics of the communities

Town	Population	Nearest dental surgery (km)	Visiting dental service	ASGC RA^a^
1	<500	248	Public dentist once every 3 months; school dental van sporadic visits	RA5
2	>2000	88	Q Coal/RFDS mobile dental service twice yearly	RA4
3	<1000	87	Private dentist once a month	RA4
4	<1000	179	Public dentist once a year, Q Coal/RFDS once yearly	RA5
5	<1000	210	Private and public dentist visits once every 3 months; Mobile Aboriginal dental van once a year; school dental van sporadic visits	RA5
6	>1500	214	Private dentist once a month for 3 days; school dental van sporadic visits, Q Coal/RFDS mobile dental service twice yearly	RA4
7	<2000	200	Private dentist visits once a month; school dental van sporadic visits	RA5
8	<3000	196	Public dentist visits once a month and mobile aboriginal van once a year	RA4

^a^Australian Standard Geographical Classification- Remoteness Area (ASGR-RA). The categories used for this study were: ASGC-RA 3 (outer regional Australia), RA4 (Remote Australia) and RA5 (very remote Australia) [38]

### Recruitment

Non-dental primary care providers in selected communities, who had experience of patients with oral health problems, were recruited to the study using purposive sampling. Dental pesonnel, including dentists, dental therapists, dental assistants and dental practice managers, who were identified by the non-dental participants as persons who had previously provided dental services to patients from the communities sampled, were subsequently recruited through a snowball sampling technique. These dental personnel had communicated directly with resident primary care workers and had an understanding of the requirements for effective dental service provision. Recruitment continued until data saturation [26] was reached in the concurrent data analyses.

### Data collection

Semi-structured interviews were conducted between Oct 2013 and December 2015 with non-dental primary care providers in selected rural and remote communities in QLD and regional or fly in/ fly out dental personnel, who had both organised and delivered dental services these communities. . The interview guide consisted of questions that sought information about the participants’ professional background; the communication they had with each other and their views on strategies that could improve communication between the two professional groups. The interview guide was based on the research questions for the larger study, established from our comprehensive literature review [13] and developed iteratively by team members. The guide was then piloted with a rural dentist and a pharmacist. Some changes were required to the interview guide to make it more specific to dental personnel.

Dental participants were asked opening questions about their experiences of working with non-dental primary care providers. They were also asked to describe referral pathways and examples of inter-professional communications between themselves and members of the non-dental health care team. Questions included:

- To whom and how often did they communicate with non-dental primary care providers?
- What types of dental presentations and issues did these non-dental personnel commonly encounter?
- What treatments and advice did they provide for dental issues?
- How did non-dental primary care providers refer patients to them? Would the use of teleconferencing or other technology be of benefit for distance diagnosis of dental problems?

Dental participants were then asked closing questions relating to strategies to improve oral health care provision in rural and remote communities.

Two dental personnel interviews were conducted face-face at their place of work by two of the authors and a further ten interviews were conducted by telephone by a one member of the research team (JS). Each interview lasted for 30–60 min.

### Data analysis

Interviews were audio recorded and transcribed verbatim into Microsoft Word and then cross checked by two authors against audio recordings for errors. The data were then imported into QSR—NVivo V.10.0 software [27] to assist with the analysis. Two authors independently analysed the interview data using thematic analysis [28] including coding the transcripts, categorising the codes and the generation of themes. Consensus amongst all team members was reached after coding results were compared and discussed at regular meetings.

### Ethics considerations

Ethics approval for the study was granted by the Human Research Ethics Committee Network (reference H0013217). Approval was obtained from the Research Committee, Royal Flying Doctor Service (RFDS) Queensland Section, to invite dental personnel employed by the service to participate in the study. Dental personnel from government clinics, private practice and the RFDS were sent an e-mail request to participate together with an information sheet detailing the purpose of study and a consent form. Participation was voluntary and participants provided consent prior to interviews.

## Results

Fifty-seven primary care providers from these communities were interviewed. Twelve dental personnel identified by primary care providers were invited and all agreed to participate in the study. This paper reports the views of the dental participants on the relationship they had with the rural primary care providers and strategies to improve the collaboration between the two disciplines. The characteristics of the dental personnel and non-dental care participants are shown in Tables 2 and 3 respectively.

**Table 2:** Characteristics of dental participants (*n* = 12)

Dental Participant Characteristics	Number of participants
Profession	
• Dentist	8 (67%)
• Dental Assistants	2 (17%)
• Dental Therapist	1 (8%)
• Dental Practice manager	1 (8%)
Gender	
Female	5 (42%)
Male	7 (58%)
Age (years)	
• 18-30	1 (8%)
• 31-40	1 (8%)
• 41-50	3 (25%)
• Over 50	7 (59%)
Years in current practice	
• <1 month	0 (0%)
• >1 month to 12 months	1 (8%)
• >1 year to 5 years	7 (58%)
• >5 years	4 (34%)
Type of sector	
Public	9 (75%)
Private	3 (25%)
Gender	
Female	5 (42%)
Male	7 (58%)

**Table 3:** Characteristics of non-dental primary health care participants (*n* = 57)

Non-Dental Participant Characteristics	Number of participants
Profession	
• Allied Health Worker	2 (4%)
• Aboriginal Health Worker	3 (7%)
• Child Health Nurse/Nurse	7 (13%)
• Director of Nursing	7 (13%)
• General Practitioner	18 (32%)
• Pharmacist	10 (18%)
• Practice Manager	7 (13%)
• Receptionist	3 (7%)
Gender	
• Female	36 (63%)
• Male	21 (37%)
Age groups	
• 18-30	13 (23%)
• 31-40	22 (39%)
• 41-50	12 (21%)
• Over 50	10 (17%)
Years in current practice	
• <1 month	6 (11%)
• >1 month to <12 months	16 (28%)
• >1 year to 5 years	25 (44%)
• >5 years	10 (17%)
Distribution of participants based on the selected communities	
• Town 1	6 (10%)
• Town 2	9 (16%)
• Town 3	7 (12%)
• Town 4	3 (5%)
• Town 5	4 (7%)
• Town 6	5 (9%)
• Town 7	5 (9%)
• Town 8	18 (32%)

Four major themes emerged from the data: (1) Communication between dental practitioners and rural primary care providers (2) Relationships between dental and primary care providers; (3) Maintenance of professional dualism; (4) Strategies to improve interprofessional relationships (with subthemes: face to face meetings; utilisation of technology; oral health training for primary care providers; and having a community based oral health contact person).

### Communication between dental practitioners and rural primary care providers

There was a lack of communication between the dental providers who saw patients in the rural communities and primary care providers in those rural communities. Some visiting dental participants believed this was because of the high turnover of medical staff in rural areas. Rural medical staff were described as “always changing”, “not stable”, “relieving ones” and “completely different”.

*Sometimes we know who the doctors and pharmacists are and it’s a case of the one’s that we meet are not stable. So it is usually a fly in, fly out doctor that comes up and does a three to six-month stint and then they go again. (Dental Assistant 1).*

*There is often very little communication with the relieving ones [medical staff]. (Dentist 7)*

Some participants stated that the rural doctors rarely contacted them for advice and vice versa.

*They [medical professionals] never contact me and I have no need ever to contact them either. (Dentist 3)*

*Never speak to a doctor and yes that is a pity isn’t it especially in today’s life with all the technology. (Dentist 7).*

One dentist had made an offer to the doctors that they could ring him for advice but this never happened.

*I work 8.00 -5.00 and I work 4 days a week. I tell the doctors they can call me but they never have. I have told the doctors they can ring me 24 h a day anytime and I will come in. (Dentist 5)*

Two of the twelve participants mentioned that the local doctors did contact them for advice on dental issues.

*The doctors ring me for advice on dental problems and I really feel that I should upgrade to dentistry. (Dental Therapist 3)*

*The [rural] doctors ring me and I speak to them about dental issues. This is good and they tell me they know very little about teeth and they refer everything teeth related to me. (Dentist 6)*

It was not uncommon for primary care providers to be unaware of the dental service visits to their community.

*…they say “oh who are you?” Unless you have been there before and seen the doctors before they have no idea who you are or what you are doing there. (Dental Assistant 1)*

Inefficient, informal or non-existent referral pathways between the primary care and dental practitioners created a barrier to effective interprofessional communication.

*I feel isolated and very frustrated with the “system”. The Central Referral Unit seems to be the problem. The phone is manned by a person who has no dental training and so is not able to triage the seriousness of conditions for priority care (Dental Assistant 2)*

There were some examples of very good communication and referrals between the two disciplines where one mobile dental service widely advertised information on their services and posted their timetables for visits to rural communities well in advance. When the hospital communicated with the visiting dental service, patients were provided with the service needed.

*The hospital rang us and said they had a patient there complaining of a toothache and we saw them. (Dental Assistant 2)*

*… we got a few referrals from a doctor by letter. Once we had treated the patient we wrote a letter back saying the treatment had been completed. (Dentist 7)*

*Yes the DONs [Director of Nursing] ring us a lot. If we are going into [Name of a rural town], we will have someone from that hospital ring us … often it is the DON. So they are the level of stakeholders that I often get calls from. So if we are doing any patient handover then they are the ones we speak to. (Practice Manager)*

### Relationships between dental and primary care providers

It was more common for visiting dental practitioners to have contact with hospital Directors of Nursing (DON), Nurse Practitioners, allied health, administrative staff and auxiliary health care workers who were often long term and committed community members than medical practitioners.

*The most stable person is the DON at the medical practice. (Dental Assistant 2)*

*In [Name of rural community] we have a good relationship but that is mainly with the admin staff more so than the doctors themselves. (Dental Assistant 1)*

*But I really think that in most places we go to it is the DONs…The ones [hospitals] that do have doctors are on shifts and you don’t get to see them. The only contact you have is the nurse’s station and possibly the DON. So, you don’t get to see the doctor most of the time. Mostly they are the same DON’s but you do get relieving DONs. The DONs that come out of the cities to do relief have different attitudes also. There is often very little communication with the relieving ones (Dentist 7).*

However, some participants acknowledged that the relationship with nursing staff was not as amicable as it could be and there was little relationship with the doctors.

*Not always do we have a good relationship with the DON. They sometimes feel like we are stepping on their toes. He was not very impressed with us using his toilets. (Dentist 7)*

*We have no professional relationships with the doctors. None what so ever (Dentist 8)*

Dental participants felt that some primary care providers did not always appear interested in dental matters and sometimes felt reluctant to approach them to co-operate in promoting oral health to patients.

*I try not to do extractions on the last few days before we leave but that doesn’t always work because you have to deal with whatever is in front of you. So I don’t want any infections once we are gone so the only thing I tell them is, listen, if our patients come in post extraction with an infected socket, this is what you need to do and I tell them. I see that their eyes glaze over straight away and they don’t want to listen (Dentist 7)*

*We could go into pharmacies and ask them to stock products but I don’t know how far it would go. Some of them might say, “This is my pharmacy and how dare you tell me how to run it”. Some will be very receptive to it all but others would say “I’ve got super floss in here but it is not up to me to teach them how to use it because I am not a dentist”. (Dental Assistant 2)*

The relationship between doctors and dentists was recognised as having two sides, as one dentist acknowledged:

*I don’t think it is just the GPs maybe because that is what they have been exposed in the past by dentist attitudes to them. I don’t think it is one sided. I think we are ahead of the game by trying to connect with doctors but it hasn’t always been that way. (Dentist 7).*

When there was a collaboration between the visiting dental providers and primary care practitioners, some participants commented that this improved dental services which was believed to benefit patients. Some DONs collaborated with the mobile dental service by distributing pamphlets and “rounding up patients” thereby increasing the number of patients successfully referred and subsequently treated by the service.

*In some of our communities, particularly the indigenous communities we have a lot of “fail to attend”. Our worst example was [Name of rural community] and so we worked very closely with the DON and said that we needed to promote the importance of the service and obviously that those people on the list need to come in, and if they can’t make it then making sure they ring up and cancel that appointment. We have seen those numbers drastically decrease by doing that. (Practice Manager)*

### Maintenance of professional dualism

The maintenance of strong disciplinary boundaries and a siloed approach to practice was seen as a barrier to interprofessional collaboration and could work to the detriment to patients with interrelated medical and dental conditions. A separation of oral health from more general health and the mouth from the rest of the body was reflected in the comments made by some participants.

*The dentists and doctors are a little bit segregated, I have found that the doctors are busier worrying about the rest of the body than saying “open up your mouth and let’s see if there is a problem there.” (Dentist 5)*

*If a patient is sick well he goes to the doctor. I fix his teeth if he has a problem (Dentist 4)*

Another participant reported that they had asked the doctors not to look at the mouth because that was the work of a dentist rather than a medical doctor.

*I told the doctors here “Don’t even look in the mouth, that’s my job”. Doctors don’t know what to do and that is why they need me. If a patient is sick well he goes to the doctor. I fix his teeth if he has a problem. (Dentist 3)*

One participant acknowledged this professional separation had been present for some time and contributed to by both the dental and medical professions:

*I don’t think it is just the GPs maybe because that is what they have been exposed to in the past by dentist attitudes to them. I don’t think it is one sided. I think we are ahead of the game by trying to connect with doctors but it hasn’t always been that way. (Dentist 7).*

### Strategies to improve interprofessional relationships

There were a number of strategies to improve the professional relationships between the disciplines that emerged from the interview data.

#### Face to face meetings

It was believed that creating opportunities to meet and organising face to face meetings between visiting dental and rural primary care providers could improve the interprofessional communication and collaboration.

*The onus would be on the dentist to go around and meet everyone [doctors and pharmacists] and say: “look, here are my timetables. This is when I will be visiting”. To say “if anyone comes your way to let them know to see me on these particular days of the week” (Dentist 3)*

If there was opportunity for the two groups to meet, this could help start building relationships and future collaboration.

*In a lot of the little towns you might be able to say come on let’s all get together, while the dental crew are in town. When we went to [Name of the rural community] the DON there was awesome. She was rounding up patients for us and she cared and I gave her so many pamphlets and stuff and she handed them out and she was all for it. (Dental Assistant 2)*

One participant recalled how a face to face meeting with the doctor helped provided the treatment need by a patient with more complex needs:

*The only reason we had that professional relationship was because the doctor came to me with a patient who was on Warfarin and they needed some extractions and she was referred by a maxillofacial surgeon in [Name of a town] and he refused to see her because she was on the warfarin. … So the doctor came in and spoke to me and we treated her. [Later] he wrote a letter of thanks to [Name of the free mobile dental services for everyone] because he was so amazed by the service. (Dentist 8)*

It was observed that younger dental graduates tended to socialise with medical graduates and this allowed respect and relationships to be developed.

*The relationship between the medical and dental people out here is very friendly because they all know each other. Often times they are young people and they associate together. So the dentists and intern doctors live together and there is a pretty close association in that regard. The smaller the community I think the better they all know each other. (Dentist 2)*

#### Utilisation of technology

The dental participants interviewed did not think that the investment in technologies such as intra-oral cameras and video or tele-conferencing would help improve dental outcomes for patients in any significant way. Respondents did however, generally appreciated the potential of new technology and the rationale for its use:

*I can see some mileage in that. Like if someone has broken a lingual cusp on a lower 6 and there’s no decay and it is sharp on the tongue, that’s the sort of thing that you could say to the nurse to just get an emery board and rub the sharp edge off. Then tell them it will be ok until they get a chance to come in and get the tooth repaired. (Dentist 2)*

However, they were less convinced of its cost benefit, especially in the absence of reliable broadband access in the bush, and the uptake of such technology by busy practitioners.

*You can get a lot of information as a dentist from an image but the doctors out bush can’t do any of the stuff that has to be done. At the end of the day the doctors will still prescribe antibiotics and give pain relief.” (Dentist 6) It’s not just from the dentist point of view but from the doctor point of view as well. They would need to have the time to take the photo and then put it into an email and that is all extra time for them as well. They are too busy out there. (Dentist 1)*

However, the introduction of these technologies was seen as a more useful tool for dentist to dentist communications, to alleviate professional isolation from dental colleagues, and to access dental specialist opinions.

*I think in some cases this would really help to have this technology especially when you have cases of pathology. It would be good to get a second opinion as to whether to do a biopsy or whatever. (Dentist 5)*

*More of a benefit if it could be networked as a mentoring tool to talk to the young dentist from when I am home. (Dentist 6)*

#### Oral Health Training for primary care providers

Dental participants perceived a need for upskilling of primary care practitioners in dental care to improve patient outcomes and to facilitate communication and collaboration with members of the dental team.

*Basically they [doctors] need to be more educated. They don’t know or have any understanding. Like you can ask them which tooth is what and they don’t have any clue. They don’t know how many roots the tooth has or where the tooth is located. They have no idea. When I was working in the hospital in theatre and so you are associating with doctors all the time and they would have no idea of the tooth numbering system. Speaking the same dental language would be a good start at least. So back to basics first, you know even if they could say “upper left first pre-molar “or something like that it would help. Then if they could work out if it was decay, abscessing or just gums bleeding. If a patient complains of bleeding gums then the doctors could tell them it’s because they are not brushing or flossing. That is probably the biggest thing that they could do apart from acute care. (Dental Assistant 2)*

The same participant commented that assistance could be provided in the diagnosis of a dental problem over the phone if the primary care provider could accurately describe the presentation.

*If they [medical staff] want advice and if they ring me, and because I have been in this job for so long, if I ask the right questions [and they describe the right issues] I can pretty much guarantee that I can diagnose the problem over the phone. (Dental Assistant 2)*

#### Having a community based oral health contact person

The lack of communication between dental and primary carers was sometimes associated with the lack of staff who were available to notify primary care providers about the visiting dental services.

*See I don’t think they have someone out there to actually say when the dentist would be there…., there was not enough staff to do that notifying as well. (Dentist 6)*

In one community, a dental therapist participant who had been in the community for a long time was known to and trusted by the community as a contact person for most matters related to oral health.

*The doctors will contact me all the time about normal every day stuff as well, not just children. I direct them where to go with their specific problems. I have had to basically direct traffic for the last 20 odd years. The community needs a contact person for their oral health questions and because I have been around for so long they ring me and trust me to know who to contact. (Dental Therapist 3)*

## Discussion

The aims of this paper were to explore the relationships between dental practitioners and non-dental primary care providers in rural and remote Queensland and strategies to improve collaboration between these disciplines from the dental personnel perspective. The results from interviews with 12 dental participants suggested that there had been a lack of communication and collaboration between dental care and rural primary care providers in these communities. The results confirm findings from studies conducted elsewhere in the world that have explored the interprofessional relationships between dentists, doctors, pharmacists and allied health care practitioners and have generally pointed to the disappointing nature of relationship between dental and non-dental disciplines [29–33]. Poor communication, high turnover of staff, the separation of oral health from overall health and the prevalence of professional dualism were seen as contributing to the lack of collaboration. Further development and implementation of both the medical rural “generalist” program and the “advanced rural dentist” concept may assist in building the bridges necessary to close this divide and benefit the community [34, 35]. The program for rural dentists could include additional training in some specialist dental procedures and the development of skills to build better collaborations with other rural health care practitioners [34].

The transient nature of locum medical practitioners and the irregular pattern of visits to these communities by dentists tended to impede the formation strong and sustainable interprofessional relationships. As a consequence, primary care providers may therefore not have made most effective use of the dental services that were available to their communities. The strongest relationships formed between dental and non-dental personnel were often between the long term resident hospital DON, allied health care providers and, on the dental side, dental assistants (or similar) and dental practice managers. In recognition of this, strategies to address the oral health needs through fly in, fly out (FIFO) or visiting mobile dental services would benefit from utilising the network of health care professionals already embedded in these communities ie. to draw on and support the further development of professional capital within these communities. Such collaborations could improve the efficiency of visiting services, continuity of care and oral health outcomes for patients.

In order to improve the interprofessional collaboration, strategies suggested by dental participants included having regular face to face meetings between the visiting/regional dental practitioners and local primary care providers, circulating the timetables of the visiting dental practitioners to the primary care providers prior to their visit to the communities, providing oral health training to primary care providers and having an oral health contact person in the community. This ‘go to’ person would have a liaison and/or information brokerage role, linking the community with visiting services and broader oral health promotion initiatives. Establishing and maintaining effective communication and referral pathways between primary care providers, dental practitioners and the local community would help build confidence in how oral health problems can be more effectively managed and, most importantly, prevented [36].

Improved oral health training for non-dental care providers in basic and preventative dental skills would provide a basis for improved communication and referral pathways between non-dental and dental care providers. There is also a role for tele-dentistry which could be used to facilitate more effective communication between health care providers, improve access to preventative dental care and tele-consultation with dental practitioners for rural and remote patients [37]. The introduction of new technologies would however, require regular training to be provided to end-users and mechanism put in place for such technology to be regularly serviced and maintained,

A limitation of the study was the small sample size that was drawn from a remote area of only one state and may not be typical of other rural communities. Also, the study involved a cross-sectional sample in which participants were asked to recall and reflect on past experiences, consequently dental services may have changed over time.

## Conclusion

The findings from this study suggest that there was little relationship between the dental practitioners and the primary care providers in the communities studied. The interprofessional collaboration between dental care providers and rural primary care providers in the rural and remote communities in QLD could be improved by having regular face to face meetings between the groups to discuss patients and oral health promotion, providing oral health education to primary care providers, establishing and maintaining effective communication and referral pathways and exploring a broader, cross-disciplinary role for tele-dentistry.

## Abbreviations

QLD: Queensland
RFDS: Royal flying doctor service
